# Nanoencapsulated Doxorubicin Prevents Mucositis Development in Mice

**DOI:** 10.3390/pharmaceutics13071021

**Published:** 2021-07-04

**Authors:** Cristiane M. Pinto, Laila S. Horta, Amanda P. Soares, Bárbara A. Carvalho, Enio Ferreira, Eduardo B. Lages, Lucas A. M. Ferreira, André A. G. Faraco, Helton C. Santiago, Gisele A. C. Goulart

**Affiliations:** 1 Department of Pharmaceutics, Faculty of Pharmacy, Universidade Federal de Minas Gerais, Belo Horizonte 31270-901, MG, Brazil; crismp@ufmg.br (C.M.P.); ufmgamanda@gmail.com (A.P.S.); eduardoburgarelli@hotmail.com (E.B.L.); lucaufmg@gmail.com (L.A.M.F.); andre.faraco@gmail.com (A.A.G.F.); 2 Department of Biochemistry and Immunology, Universidade Federal de Minas Gerais, Belo Horizonte 31270-901, MG, Brazil; laila.horta@yahoo.com.br (L.S.H.); heltonsantiago@icb.ufmg.br (H.C.S.); 3 Department of General Pathology, Biological Science Institute, Universidade Federal de Minas Gerais, Belo Horizonte 31270-901, MG, Brazil; barbaraandradedecarvalho@yahoo.com.br (B.A.C.); eniofvet@hotmail.com (E.F.)

**Keywords:** nanostructured lipid carriers, doxorubicin, mucositis

## Abstract

Doxorubicin (DOX), a chemotherapy drug successfully used in the therapy of various types of cancer, is currently associated with the mucositis development, an inflammation that can cause ulcerative lesions in the mucosa of the gastrointestinal tract, abdominal pain and secondary infections. To increase the safety of the chemotherapy, we loaded DOX into nanostructured lipid carriers (NLCs). The NLC–DOX was characterized by HPLC, DLS, NTA, Zeta potential, FTIR, DSC, TEM and cryogenic-TEM. The ability of NLC–DOX to control the DOX release was evaluated through in vitro release studies. Moreover, the effect of NLC–DOX on intestinal mucosa was compared to a free DOX solution in C57BL/6 mice. The NLC–DOX showed spherical shape, high drug encapsulation efficiency (84.8 ± 4.6%), high drug loading (55.2 ± 3.4 mg/g) and low average diameter (66.0–78.8 nm). The DSC and FTIR analyses showed high interaction between the NLC components, resulting in controlled drug release. Treatment with NLC–DOX attenuated DOX-induced mucositis in mice, improving shortening on villus height and crypt depth, decreased inflammatory parameters, preserved intestinal permeability and increased expression of tight junctions (ZO-1 and Ocludin). These results indicated that encapsulation of DOX in NLCs is viable and reduces the drug toxicity to mucosal structures.

## 1. Introduction

Cancer incidence and deaths are increasing worldwide. According to the World Health Organization (WHO), the number of cancer deaths is expected to double between 2018 and 2040 [1]. Part of these deaths will be attributed to adverse events caused by the cancer treatment itself, or to the interruption of the treatment caused by such adverse events. One way to minimize these deaths and ameliorate the quality of life of patients is to develop treatments with improved efficacy and decreased toxicity. Among the efforts to improve cancer treatment, the encapsulation of active pharmaceutical ingredients (APIs) in nanocarriers, which can reduce the toxicity of the APIs used in cancer therapy, is one of the most studied options [2,3,4].

Mucositis, an inflammatory condition of the gastrointestinal tract (GIT) mucosa, is one of the most common and uncomfortable adverse reactions during cancer treatment, affecting at least 40% of patients. This condition can lead to ulcerative lesions, causing pain and discomfort to the patients, interfering directly in their quality of life and adherence to treatment. It can also lead to secondary infections and other complications, such as septicemia, due to increased GIT permeability [5,6,7].

Aiming to improve the safety of the treatment with chemotherapeutic agents, we proposed its encapsulation in nanostructured lipid carriers (NLCs), using doxorubicin (DOX) as a model. DOX is an API widely used in cancer therapy, being effective in treatment of several types of cancer, such as leukemia, Hodgkin’s and non-Hodgkin’s lymphomas and breast cancer [8]. To be used in the clinic, DOX is formulated as a hydrochloride salt dissolved in an aqueous solution for intravenous injection [9], being rapidly distributed into tissues [10,11].

Although DOX is subject to many studies, the mechanism of action is still considered unclear. It has been suggested that DOX can intercalate dsDNA and promote their break [12] and also impair the action of Topoisomerase II [12,13]. In addition, DOX has also been regarded as promoting oxidative stress when coupled with iron [14]. All of these mechanisms lead to DNA breakage or damage and inhibition of DNA and RNA synthesis, which compromise cell viability and induce a plethora of drug-associated adverse events, including cardiotoxicity and mucositis. These adverse events can lead to treatment interruption, in addition to increased treatment costs and mortality ratio [15,16,17,18,19]. The clinical approach to DOX-associated toxicities is updated regularly [20]. However, there is still a pressing need for fewer toxic drugs formulation and presentation. In order to circumvent the adverse effects of DOX and improve its efficacy, different drug delivery systems have been developed [21,22,23,24,25,26,27,28,29,30]. Among lipid-based systems, Doxil ^®^ and Myocet ^®^ are liposomal commercial formulations, which improved the safety profile of DOX in comparison with free DOX. However, mucositis remains one of the main recurring adverse effects [31,32]. Therefore, a formulation that will spare intestinal mucosa during chemotherapy is still a pressing necessity for cancer patients.

As liposomes, solid lipid nanoparticles (SLN) and NLC can improve bioavailability and control drug release [27,28,33], but they present advantages, as production without organic solvents and using simple and easily scale-up techniques, which turns the process simpler, safer and cheaper [34,35]. Different from SLN, NLCs are drug delivery systems composed of a mixture of solid and liquid lipids, thus forming a solid lipid matrix containing imperfections in which the APIs can be loaded [36,37]. Müller et al. [38] first described the NLCs in 2002 and after that, many studies have been carried out with different APIs, including that used in chemotherapy [39,40]. However, due to the hydrophilic characteristic of the DOX hydrochloride salt, its encapsulation in lipid nanocarriers is low [41,42] and the ion pair strategy has been used to enhance DOX incorporation in the lipid matrix. Through this strategy, DOX forms an ion pair with a lipophilic anion, improving its lipophilicity and the affinity for the lipid matrix. This strategy has improved the efficacy of DOX, as well as reduced its toxicity in different lipid-based systems [33,41,43,44,45,46,47,48,49,50,51,52,53,54,55,56].

Although there is an increasing interest in the use of NLC in cancer treatment, the use of NLCs to mitigate or prevent mucositis is poorly investigated, especially using DOX. Therefore, the aim of this study was to design and to characterize an NLC loaded with DOX (NLC–DOX), using the ion pair strategy, and compare its safety to free DOX in a mouse model of DOX-induced mucositis, which is an established model to assess the DOX-induced mucositis [19]. We found that NLC–DOX were less toxic to the animals’ intestines. This study was the first to assess the impact of DOX encapsulation in NLC as a strategy to reduce the development of mucositis.

## 2. Materials and Methods

### 2.1. Materials

Doxorubicin hydrochloride (DOX) was kindly provided by ACIC Chemicals (Ontario, Canada). Oleic acid was purchased from Sigma-Aldrich (St. Louis, MO, USA). Triethanolamine (TEA) was obtained from Merck (Darmstadt, Germany). Lipoid MCT was purchased from Lipoid GmbH (Ludiwigshafen, Germany). Monooleate of sorbitan (Span 80 ^®^) and monooleate of sorbitan ethoxylated (Super Refined Polysorbate 80 ^®^) was provided by Croda Inc. (Edison, NJ, USA). Glyceryl behenate (Compritol 888 ATO ^®^) was provided by Gattefossé (Saint Priest, France). All other chemicals were of analytical grade.

### 2.2. Preparation of NLC–DOX and Free DOX Solution

NLCs were prepared by the hot melting homogenization method, as previously described [27]. During the development of the NLC–DOX we evaluated different proportions of liquid (MCT) and solid lipids (Compritol 888 ATO ^®^), as well as the surfactants Tween 80 ^®^ and Span 80 ^®^. The better formulation was obtained by using 60:40 of solid:liquid lipids and a mixture 80:20 of Tween 80 ^®^:Span 80 ^®^. Briefly, batches (10 mL) were prepared containing an oil and aqueous phases. The oil phase (OP) was composed of 120 mg of Compritol 888 ATO ^®^, 80 mg of MCT, 200 mg of Tween 80 ^®^, 50 mg of Span 80 ^®^, 15 mg of DOX, 12 mg of TEA and 30 mg of oleic acid. The aqueous phase (AP) was composed of distilled water. The OP and AP were heated separately to 80 °C and then gently mixed under constant agitation, at 8000 rpm, with an Ultra Turrax T-25 homogenizer (Ika Labortechnik, Staufen, Germany) at 80 °C. The emulsion formed was homogenized for 10 min on ultrasound with high power probe (21% amplitude, Ultra-cell 750 W, Sonics Materials Inc., USA). The pH of the NLC–DOX was adjusted to 6.5 with a solution of 0.1 M HCl and the formulations were stored at 4 °C, protected from light in a nitrogen atmosphere. The non-encapsulated DOX solution (free DOX), in the same concentration of NLC–DOX (1.5 mg/mL), was prepared diluting DOX in distilled water.

### 2.3. Particle Size Analysis and Zeta Potential

The hydrodynamic diameter of the NLC–DOX was determined by unimodal analysis through dynamic light scattering (DLS), and the data reported were Z-average, evaluated as the intensity. The hydrodynamic diameter, polydispersity index (PDI) and Zeta potential of the NLC–DOX were determined by dynamic scattering of light and electrophoretic mobility, using a Zetasizer Nano-ZS90 (Malvern Instruments; England) at a fixed angle of 90° and temperature of 25 °C. For DLS, the NLC-DOX was diluted (1:100) in ultrapure water previously filtered through cellulose ester membrane with 0.45 µm pore diameter (HAWPO4700, Merck Millipore, Burlington, MA, USA). All measurements were performed in triplicate.

### 2.4. Nanoparticle Tracking Analysis (NTA)

NTA experiments were performed by using the NanoSight NS300 (Malvern Instruments; Salisbury, England). After appropriate dilution of the NLC-DOX in ultrapure water, the sample was introduced into the NanoSight sample chamber with a disposable syringe. The samples were measured at room temperature for 60 s with automatic detection. The software used for capturing and analyzing the data was the NanoSight NS300 (Malvern Instruments, Salisbury, England).

### 2.5. Drug Encapsulation Efficiency (EE) and Drug Loading (DL)

The encapsulation efficiency (EE) and drug loading (DL) of DOX in NLC were determined by an ultrafiltration method, using centrifugal devices (Amicon^®^ Ultra−0.5 mL 100 k; Millipore, USA). To eliminate the binding of DOX to the devices, a pretreating of the filters was performed as previously described [45]. The devices were soaked in a passivating solution (Tween 20 ^®^, 5% *w*/*v*), maintained overnight at room temperature and washed with distilled water prior to use. The EE and DL were calculated by using the following Equations (1) and (2), respectively:

EE% = [(CT − CAP)/CT] × 100(1)

DL (mg/g) = WDL/WNP(2)

where CT = total DOX concentration in NLC; CAP = DOX concentration in aqueous phase (non-encapsulated drug); WDL = mg of drug loaded in NLC and WNP = g of lipids.

The CT, CAP and WDL were evaluated as described by Mussi et al. [27]. The CT was analyzed by dissolving an aliquot of the NLC dispersion in a mixture of tetrahydrofuran (THF)/methanol (MeOH) 40:60 *v*/*v*, followed by centrifugation (10 min at 2400 g) and analysis of the supernatant by UV-vis spectrophotometry at 480 nm (UV-Vis Evolution 201; ThermoFisher, Shanghai, China). The CAP was evaluated from an aliquot of the aqueous phase separated from the NLC dispersion by ultrafiltration (10 min at 2400 g), dilution with THF/MeOH and analysis by UV-vis. The WDL was derived by using the calculated EE × total (mg) of DOX added. The method for DOX quantification was previously validated. The five-point linear regression analysis resulted in the following linear equation: y = 0.01930X–0.00615 (R^2^ = 0.9915).

### 2.6. Differential Scanning Calorimetry

Differential scanning calorimetry (DSC) analysis of formulation components (DOX and Compritol 888 ATO) and NLC-DOX were performed by using a DSC 2910 differential scanning calorimeter (TA Instruments, New Castle, DE, USA). For DSC measurements, a scan rate of 10 °C/min was used at a temperature range of 0–400 °C, under a nitrogen purge. NLC-DOX was lyophilized prior to analysis with the use of a freeze-drier (Modulyod-115, ThermoFisher, Asheville, NC, USA; Pump Edwards E2M18, West Sussex, UK). After freeze-drying, the NLCs sample, DOX and Compritol were placed directly in aluminum pans for analysis (approximately 7 mg of lipid).

### 2.7. ATR–FTIR Analysis

Attenuated total reflection–Fourier transform infrared (ATR–FTIR) spectra of NLC-DOX and raw materials (DOX and Compritol) were recorded within films on a Perkin Elmer FTIR spectrometer (Model Spectrum One).

### 2.8. Transmission Electron Microscopy (TEM) and Cryogenic TEM (Cryo-TEM)

The morphology of the NLC-DOX was evaluated by TEM. Negatively stained samples were prepared by spreading 3 µL of the NLC-DOX dispersion (diluted 1:25) onto a copper grid coated with a Lacey carbon film. After 1 min of adsorption, excess liquid was blotted off with filter paper and the grids were placed on a droplet of 2% (*w*/*v*) aqueous uranyl acetate and drained off after 1 min. The dried specimens were examined by using a 120 kV electron microscope (Tecnai-G2-Spirit-FEI/Quanta microscope; Philips, Eindhoven, Netherlands). The analyses involving TEM were conducted at the Microscopy Center of UFMG (http://www.microscopia.ufmg.br accessed on 26 August 2020).

Cryo-TEM micrographs of NLC-DOX were obtained by using a TEM (Tecnai Spirit G2-12 Biotwin-FEI, Brno, Czech Republic) operated at 120 kV. Images were recorded on the CCD Eagle (FEI), using the Serial EM software. Prior to use, the 300 mesh lacey carbon coated copper grids (EMS) were subjected to the glow discharging process with argon gas at a current of 9.2 mA for 25 s. Cryo-TEM samples were prepared by plunge freezing (Leica EM GP2). For each sample, a drop of 3 µL was placed on the carbon side of the grid, blotted for 5 s and immediately frozen in liquid ethane, and then kept in liquid nitrogen until cryo-TEM. The samples were mounted on Fischione cryo holder (Model 2550) and analyzed in the TEM at −175 °C. Pictures were processed by using ImageJ software (National Institutes of Health, Betesda, MD, USA). The analyses involving cryo-TEM were conducted at the Microscopy Center of UFMG (http://www.microscopia.ufmg.br accessed on 16 December 2020).

All the acquired images were uploaded to accessible imaging software (Image J, US National Institutes of Health, Bethesda, MD, USA). The particles were measured from images obtained from three independent NLC-DOX formulations. Before measuring the size, each photo was precisely dimensioned, using a calibrated ruler. The normal distribution of the size was analyzed by using the Shapiro–Wilk test, assuming a 95% confidence level.

### 2.9. In Vitro DOX Release

The release of DOX from NLC-DOX was performed by using the dialysis method. Dialysis bags with a cutoff size of 14,000 Da and diameter of 21 mm (cellulose ester membrane; Sigma-Aldrich, St. Louis, MO, USA) were filled with 2 mL of NLC-DOX, sealed and incubated with 50 mL of phosphate-buffered saline (PBS) pH 7.4, for 72 h at 37 °C, using shaking stirring (IKA ^®^ KS 4000 i control; Werke, Germany) at 120 rpm. An aqueous solution of non-encapsulated DOX (free DOX) at the same concentration (1.5 mg/mL) was used as a control. At different predetermined time intervals (1, 2, 4, 8, 24, 48 and 72 h), 2 mL of release media were taken out and replenished with an equal volume of fresh PBS. The amount of released DOX was measured by UV-vis spectrophotometry as described above. The values were plotted as a cumulative percentage of DOX release. Released DOX (%) = RF/CD × 100. Where RF = released fraction of DOX to the external medium, and CD = initial concentration of DOX inside the dialysis bag.

The regression analysis of the cumulative release of DOX from NLC-DOX and free DOX was evaluated. Different kinetic models were evaluated: zero order (Q_t_ = Q_0_ + K_0_t), first order (ln Q_t_ = ln Q_0_ +K_1_t), Higuchi (Q_t_ = K_h_t^1/2^), Hixson–Crowell (Q_0_^1/3^ − Q_t_^1/3^ = K_s_t) and Korsmeyer–Peppas (Q_t_/Q_∞_ = K_k_t^n^); where Q_t_ is the amount of drug released at time t; Q_0_ is the amount of the drug in the solution at time zero and K is constant of release kinetics. Determination coefficient (R^2^) was used as a criterion to define the best release kinetics model [57,58]. All experiments were conducted in triplicate.

### 2.10. Animals and Experimental Groups

All procedures were approved by the Ethics Committee for Animal Experimentation at the Federal University of Minas Gerais (UFMG) (Protocol n° 331/2012, 22/11/2012). C57BL/6 female mice, free of specific pathogens, 6–8 weeks old, were obtained at the Central Vivarium of UFMG, weighed and randomly divided into three experimental groups. The animals were kept in collective cages with a maximum of five individuals per cage, with water and diet offered freely. The environmental conditions were controlled, using a 12-h light/dark cycle and temperature around 28 °C. The animals in the control group (Saline) received 100 μL of saline solution by intraperitoneal administration (i.p.), while the groups free DOX and NLC-DOX received DOX at 10 mg/kg by i.p. After three days, at the peak of intestinal inflammation, the animals were anesthetized with i.p. injection of ketamine (100 mg/kg) and xylazine (5 mg/kg) and blood was collected followed by cervical dislocation. The small intestine of animals was collected and divided into parts (duodenum, distal jejunum, proximal jejunum and ileum). The distal ileum portion (approximately 1 cm) was frozen at −80 °C for evaluation of cytokines. The rest of the ileum was used for histological and morphometric analysis.

### 2.11. Histological Analysis

Samples of the ileum of the C57BL/6 mice were fixed in formaldehyde (10%) and processed for histological analysis. The cuts obtained were stained with hematoxylin and eosin for morphological evaluation. The morphometry of villus height and crypt depth was obtained from photos of histological slides, which were analyzed by using the ImageJ software (National Institutes of Health, Betesda, MD, USA). Ten random fields per animal were computed, with tissue integrity as a limiting factor.

### 2.12. Analysis of Gene Expression

To evaluate the expression of cytokines in the small intestine, 1 cm of the distal ileum was extracted, using Trizol (TRI reagent, Sigma-Aldrich) following acid–alcohol affinity and kept at −80 °C until use. One microgram of total RNA was reverse-transcribed by using M-MLV RT (Promega). Gene expression was performed on n aABI PRISM 7900 sequence detection system (Applied Biosystems), using HOT FIREPol Eva Green qPCR Supermix (Solis BioDyne) according to manufacturer’s instructions. Briefly, samples were exposed to 95 °C for 12 min and then subjected to 40 cycles of 95 °C for 15 s and 60 °C for 30 s. At the end of the cycling, a melting curve was performed to evaluate primer specificity. Expression of the target genes (Table 1) were normalized with GAPDH (glyceraldehyde 3- phosphate dehydrogenase), using 2-ΔΔCt. All primers were obtained at Harvard Primer Bank.

### 2.13. Assessment of Intestinal Permeability

Intestinal permeability was determined by measuring the radioactivity diffused in the blood after administration of diethylenetriamine-pentacetic acid (DTPA) with technetium 99 m (99 mTc) [59]. Three days after administration of saline, free DOX and NLC-DOX, all mice received, by gavage, 0.1 mL of DTPA solution conjugated to 18.5 mebequerel (MBq) of 99 mTc. Four hours later, all animals were anesthetized to collect 300μL of blood in EDTA (ethylenediamine tetraacetic acid), which was placed in appropriate tubes to determine radioactivity. The data were obtained in % of dose, using the following equation: % Dose = (cpm of blood/cpm of administered dose) × 100, where cpm represents counts per minute.

### 2.14. Statistical Analysis

Statistical analyses were performed by using GraphPad Prism^TM^ (version 8.2). Data were expressed as mean ± standard deviation (SD). Data normality was assessed by using the Shapiro–Wilk test. To analyze the difference in the DOX release profile and in the body weight variation of mice, two-way analysis of variance (ANOVA) was used, followed by Tukey’s post-test. To compare the morphological alterations among groups, the cytokine expression by real time, intestinal permeability, occludin and ZO1, one-way ANOVA was used, followed by the Tukey’s post-test. For all analyses, a significance level of 0.05 was used.

## 3. Results

### 3.1. Characterization of the Developed NLC-DOX

**Figure 1:**
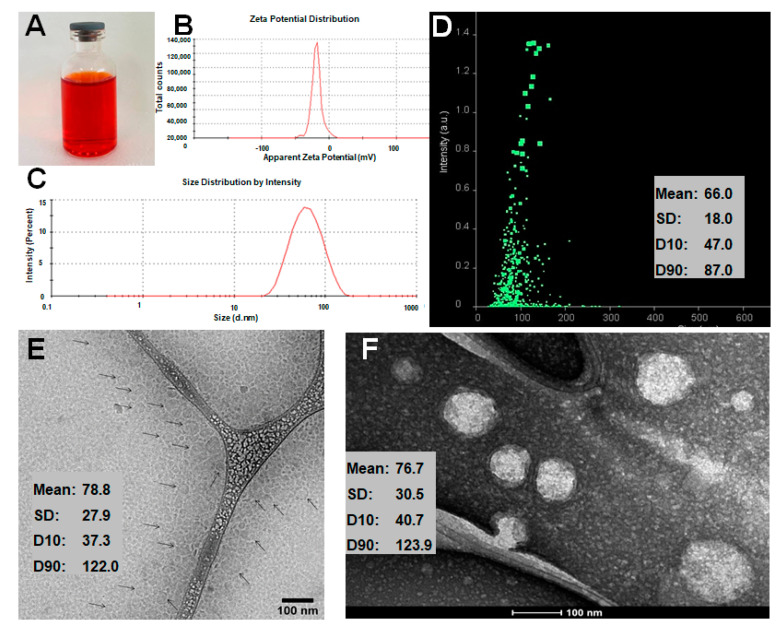
Characteristics of NLC-DOX. Image of NLC-DOX formulation (**A**); Zeta potential distribution determined by dynamic scattering of light and electrophoretic mobility (**B**); particle size distribution determined by DLS (**C**); particle size distribution determined by NTA (**D**); cryo-TEM image (**E**); and TEM image of NLC-DOX (**F**). Abbreviations: DOX, doxorubicin; NLC, nanostructured lipid carrier; DLS, dynamic light scattering; NTA, nanoparticle tracking analysis; TEM, transmission electronic microscopy; SD, standard deviation; D10, the size point below which 10% of NLC-DOX is contained; D90, the size point below which 90% of NLC-DOX is contained.

**Figure 2:**
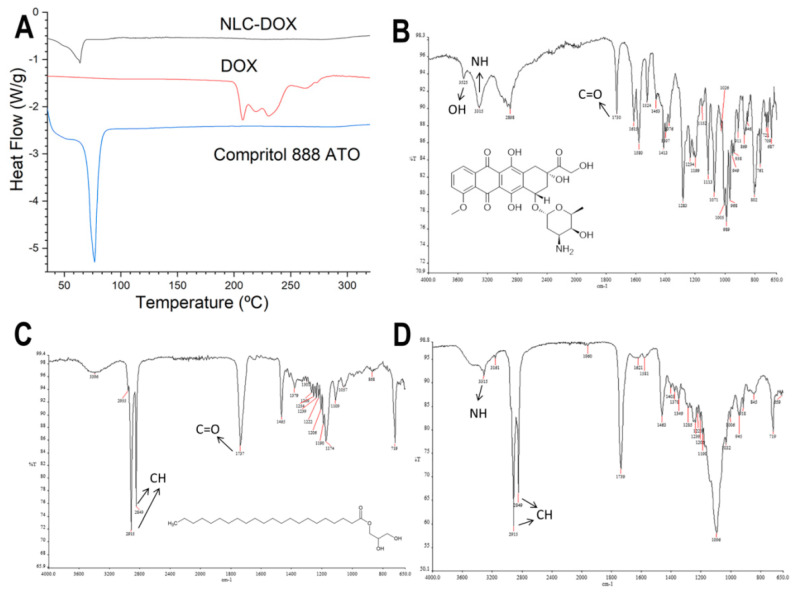
Characterization of the NLC-DOX formulation and its main components by differential scanning calorimetry (DSC) and attenuated total reflection–Fourier transform infrared (ATR-FTIR). DSC curves of NLC-DOX, pure DOX and Compritol 888 ATO ^®^ (**A**); ATR-FTIT spectra of DOX (**B**), Compritol 888 ATO ^®^ (**C**) and NLC-DOX (**D**). The arrows indicate the main absorption regions of the functional groups. Abbreviations: DOX, doxorubicin; NLC, nanostructured lipid carrier.

NLC-DOX was found to be a clear and homogeneous red suspension (Figure 1A) and could efficiently incorporate DOX. The total DOX content in the developed NLC-DOX was 99.8 ± 1.5%, with high encapsulation efficiency (84.8 ± 4.6%). The Zeta potential was −18.3 ± 5.1 mV (Figure 1B) and the average size and PDI obtained by DLS were, respectively, 69.7 ± 6.6 nm (Figure 2C) and 0.29 ± 0.07. The average size determined by NTA (Figure 1D) was slightly smaller (66.0 ± 18 nm). The size and the morphology of NLC-DOX was also evaluated by Cryo-TEM and TEM (Figure 1E,F, respectively). The TEM (76.7 ± 30 nm) and Cryo-TEM (78.8 ± 28 nm) images showed particles homogeneous with spherical shape and normal distribution (Shapiro–Wilk test) at a confidence level of 95%.

The thermal behavior of the NLC-DOX and its main components (DOX and the solid lipid Compritol 888 ATO) was evaluated by DSC (Figure 2A). The thermogram of pure DOX showed three endothermic peaks at temperatures between 210 and 240 °C, while the thermogram of pure Compritol 888 ATO showed only one narrow, symmetrical endotherm peak with the melt temperature of 69.79 °C. The NLC-DOX also showed only one endothermic peak at 63.85 °C, corresponding to its solid lipid matrix (Compritol 888 ATO). However, a slight decrease in the melting point (69.79 °C for pure lipid versus 63.85 °C for NLC-DOX), as well as a broadening of this peak was observed. Moreover, the presence of the endothermal melting peak of DOX was not observed in the thermogram of NLC-DOX, suggesting a high affinity of the DOX to the lipid matrix.

The FTIR spectra of DOX, NLC-DOX and Compritol 888 ATO ^®^, the lipid matrix of the NLC-DOX, are presented in Figure 2B–D. FTIR analysis is usually used to determine the interaction between drugs and excipients, observing the spectrum of functional groups [60]. The spectrum of the DOX (Figure 2B) shows the characteristic bands of the drug: 3525 and 3315 cm^−1^ bands, which are attributed to the O-H and a N-H group stretching vibration; and a band at 1730 cm^−1^, due to stretching of C=O, which are in accordance of those described in the literature [60,61]. The FTIR spectra of Compritol 888 ATO ^®^ showed characteristic peaks in 2915 cm^−1^ (stretching of C-H), 1737 cm^−1^ (stretching of C=O) and 1465 cm^−1^ (bending of C-H). Similar results were found by [62,63]. In the spectrum of NLC-DOX, it is also possible to observe the band from N-H group at 3315 cm^−1^, that can be attributed to DOX’s molecule; and two broad bands at 2915 and 2849 cm^−1^, that can be attributed to the stretching vibration of C-H and C-H_2_ groups from Compritol 888 ATO ^®^.

### 3.2. In Vitro DOX Release

**Figure 3:**
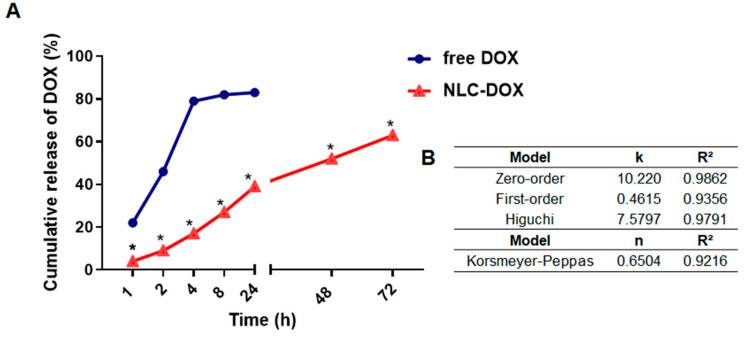
In vitro drug release profile of DOX from NLC-DOX and free DOX (**A**). Kinetic models applied to the release of DOX from NLC-DOX (**B**). * *p* < 0.0001. Data were presented as mean ± SD (n = 3). Abbreviations: DOX, doxorubicin; NLC, nanostructured lipid carrier; k, kinetic constant; n, diffusion exponent.

The in vitro DOX release from NLC-DOX and free DOX was investigated by using dialysis bags in PBS buffer pH 7.4 (37 °C ± 0.5). As can be seen in Figure 3A, free DOX exhibited a rapid release of 80% of drug within 4 h, whereas the release profile of NLC-DOX indicated a sustained pattern without a burst release. The mechanism of release was evaluated by using four different kinetic models (zero-order, first-order, Higuchi and Korsmeyer–Peppas) and the regression coefficient value (R^2^) was used to choose the model that best fit the data. The zero-order model presented the higher R^2^ (Figure 3B), suggesting a process of constant drug release from the NLC system, independent of the DOX’s concentration. This is ideal behavior for a dosage form and leads to minimum fluctuations in drug plasma levels [64].

### 3.3. NLC-DOX Treatment Prevents Weight Loss in C57BL/6 Mice

**Figure 4:**
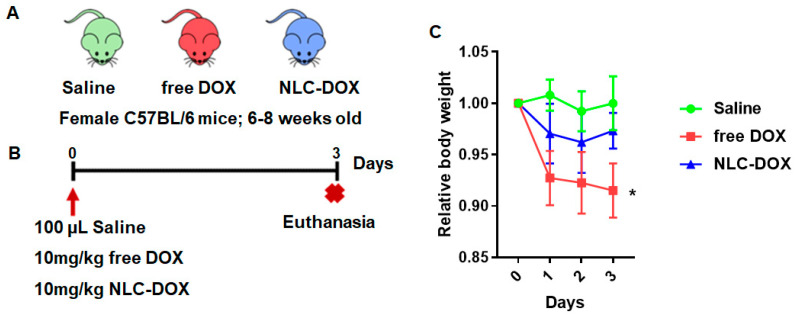
NLC-DOX prevents DOX-induced weight loss. (**A**) C57BL/6 mice were randomly allocated to 3 groups, Saline, free DOX and NLC-DOX (n = 5 animals/group). (**B**) Animals were injected with free DOX, NLC-DOX or saline and followed for 3 days. (**C**) Weight variation of the animals during the 3-day follow-up. The animals were weighed daily, the first weighing was performed on day zero of the protocol and the average weight per group analyzed was considered for analysis. Statistical analysis was performed by using the ANOVA test, followed by the Tukey test. * *p* < 0.05. N = 20 animals per group. Data are a pool from 4 experiments performed. Abbreviations: DOX, doxorubicin; NLC, nanostructured lipid carrier.

To compare the evolution of mucositis between free DOX and NLC-DOX, we used a DOX-induced mouse model of mucositis [19]. C57BL/6 female mice (6–8 weeks) were randomly divided into three groups (Figure 4A) and inoculated i.p. with free DOX (10 mg/kg), NLC-DOX (10 mg/kg) or saline solution for the Saline group (Figure 4B). Animals treated with both DOX formulations presented weight loss in the first days of mucositis induction, which was attenuated by DOX encapsulation (NLC-DOX group) (Figure 4C). However, blood in the stool or diarrhea, common features of intestinal inflammation, were not observed in either group.

### 3.4. NLC-DOX Preserves Bowel Architecture, Prevents Mucositis-Compatible Ulcerations and Improves Intestinal Permeability

**Figure 5:**
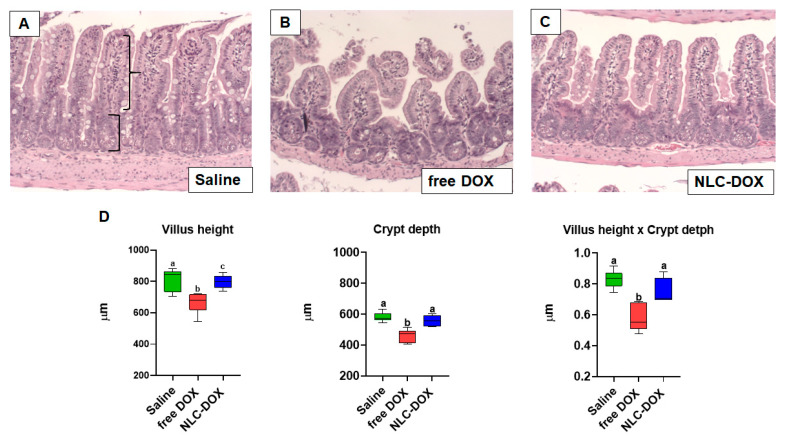
NLC-DOX prevented DOX-induced mucositis-associated intestinal morphological alterations. (**A**–**C**). Representative histological slides of ileum of Saline, free DOX and NLC-DOX treated animals (20× magnification). (**D**) Histomorphological analysis of jejune and ileum was used to quantify the height of the villus (represented by curly bracket), depth of the crypts (represented by square bracket). Statistical analysis was performed by using the ANOVA test, followed by the Tukey test. Different letters represent *p* < 0.05. N = 10 animals per group. Data are a pool from 2 experiments performed. Abbreviations: DOX, doxorubicin; NLC, nanostructured lipid carrier.

The treatment with free DOX induced changes in intestinal architecture typical of mucositis. Free DOX treated animals displayed multifocal inflammatory areas in jejune and ileum with important infiltration of mononuclear cells associated with reduced villus height, reduced crypt depth and thinning of the muscular layer (Figure 5). Ileum showed more prominent pathological changes. All of these features were improved, or even prevented, in animals treated with NLC-DOX.

**Figure 6:**
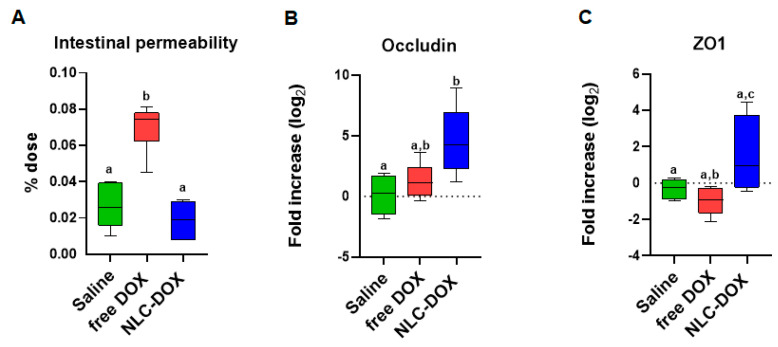
Intestinal permeability was preserved in NLC-DOX treatment. (**A**) Intestinal permeability by DTPA-Tc99m and mRNA expression of (**B**) Occludin and (**C**) ZO1 by real time RT-PCR in the ileum were evaluated 3 days after injection of saline, free DOX or NLC-DOX. Statistical analysis was performed by using the ANOVA test, followed by the Tukey test. Different letters represent *p* < 0.05. N = 10 animals per group. Data are a pool from 2 experiments performed. Abbreviations: DOX, doxorubicin; NLC, nanostructured lipid carrier.

Mucositis is often associated with compromised intestinal function allowing bacteria translocation due to increased permeability. We found that associated with the inflammation and changes in intestinal architecture induced by free DOX, intestinal permeability was also increased in free DOX treated animals (Figure 6A), but totally preserved in animals treated with NLC-DOX and Saline. In addition, the maintenance of intestinal permeability in NLC-DOX was associated with increased expression of mRNA for tight junction proteins such as Occludin (Figure 6B) and ZO1 (Figure 6C) when compared to Saline or free DOX.

### 3.5. NLC-DOX Formulation Maintains Cytokine Expression

**Figure 7:**
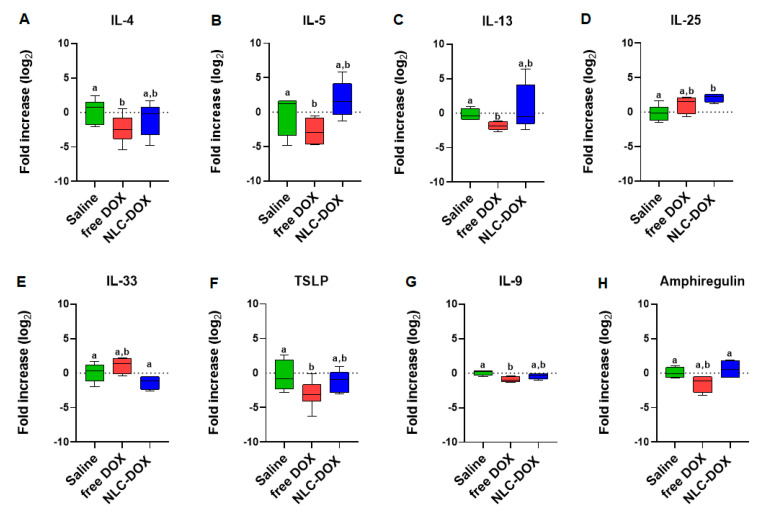
Cytokine expression by real time RT-PCR of (**A**) IL-4, (**B**) IL-5, (**C**) IL-13, (**D**) IL-25, (**E**) IL-33, (**F**) TSLP, (**G**) IL-9 and (**H**) Amphiregulin. Statistical analysis was performed by using the ANOVA test, followed by the Tukey test. Different letters represent *p* < 0.05. N = 15 animals per group. Data are a pool from 3 experiments performed. Abbreviations: DOX, doxorubicin; NLC, nanostructured lipid carriers.

To gain insight about the quality of the inflammatory response triggered by DOX treatment, we evaluated signature cytokines associated with mucositis. As can be observed in Figure 7, the expression of cytokines was usually decreased in free DOX group when compared to saline group (IL-4, IL-13, TSLP and IL-9, *p* < 0.05), possibly associated to the reduced tissue viability associated to the drug cytotoxicity. However, in the group treated with NLC-DOX, the expression of cytokines was similar to the saline group, except for IL-25, a cytokine associated with intestinal protection [65], which was increased.

## 4. Discussion

DOX is a chemotherapy drug of the anthracycline class widely used in clinical practice [66,67]. Although DOX has a relevant role in cancer therapy, its adverse effects, such as mucositis, have an important impact in the treatment and quality of life of patients [18,19]. To overcome the intense mucositis induced by DOX treatment, we proposed the loading of DOX in NLCs. This study was the first that evaluated the impact of DOX encapsulation in the lipid matrix of NLCs as a strategy to reduce mucositis.

To be used in the clinic, DOX is formulated as a hydrochloride salt, which is freely soluble in water. Therefore, its encapsulation in lipid nanocarriers is not an easy issue, being usually low [41,42]. To enhance its encapsulation in the lipid nanocarriers, the ion-pair strategy was used. Through this strategy, DOX forms an ion pair with a lipophilic anion, improving its lipophilicity and the affinity for the lipid matrix. This strategy is well described in the literature and has been used in several studies that utilized different counter ions to improve the DOX lipophilicity and its affinity for different lipid-based systems [33,41,43,44,45,46,47,48,49,50,51,52,53,54,55,56]. In the present study, OA was used as the counter ion. During the NLC production, the OA reacts with the positive electrostatic charge localized at the protonated amino nitrogen of the doxorubicin base (pKa = 9.53), forming an ion pair and improving the DOX affinity for the lipid matrix. The formation of the ion pair between OA and DOX is well described in the literature [28,51].

The developed NLC-DOX presented high encapsulation efficiency (84.8 ± 4.6%) and drug loading (55.2 ± 3.4 mg/g), minimizing the amount of soluble DOX in the external phase of the nanoparticles suspension. The obtained encapsulation efficiency (84.8 ± 4.6% EE) is in accordance with the results described by other authors (99.15–74.18% EE). However, our data concerning the drug loading (55.2 ± 3.4 mg/g) were much higher than those previously published (31 to 10.1 mg/g) [27,68,69,70]. Other parameters, such as morphology (spherical shape), size (66–78.8 nm), PDI (0.29 ± 0.07) and Zeta potential (−18.3 ± 5.1 mV), obtained for the developed NLC-DOX are in accordance with the literature data for parenteral formulations [27,67,68]. The negative Zeta potential can be explained by the presence of OA and non-ionic surfactants (Polysorbate 80 and Span 80) used in NLC-DOX [71,72]. The average size obtained from NTA analysis was slightly smaller (66 ± 18 nm), but very close to the average size provided for the DLS method (69.7 ± 6.6 nm). The smaller average size found for the NTA method is in accordance with the literature and corroborated the monodisperse distribution (low PDI values) measured by DLS [73,74]. Regarding the error bars of the size distribution, they are smaller with DLS, which is a consequence of the large amount of statistical data collected by this method when compared to NTA. In the NTA method, the size distributions are practically the same, but the software sometimes detects slightly more or slightly less particles between each measurement of the same sample [74].

In contrast to DLS and NTA techniques, electron microscopy—especially cryo-TEM—allows direct observation of individual NLC-DOX particles. The values obtained from TEM (76.7 ± 30 nm) and cryo-TEM (78.8 ± 28 nm) images are in a reasonable agreement with DLS and NTA analysis. Precise measurements of NLC-DOX particles from TEM and cryo-TEM images, accompanied by data provided by DLS and NTA, allowed us to obtain a more real particle size average of the developed NLC-DOX, which is a very important feature for parenteral drug delivery systems [75].

The high encapsulation efficiency and drug loading of DOX in the lipid matrix of NLCs should be the result of the intense interactions between the formulation components. In the DSC analysis, the DOX thermogram showed three endothermic melting peaks (206.8, 218.4 and 237.3 °C), which are in accordance with those described in the literature [76,77]. However, any peak of DOX was observed in the NLC-DOX thermogram, suggesting a high interaction of DOX with the lipid matrix of NLCs. This high affinity for the lipid matrix of NLCs can be the result of the ion pair formation between DOX and oleic acid (OA). The formation of an ion pair between DOX and OA has been evaluated by different authors as a strategy to improve the encapsulation efficiency of DOX in lipid nanoparticles without limiting its efficacy [27,45,78].

Another important result to note in the DSC thermogram of NLC-DOX is the characteristic endothermic peak of Compritol 888 ATO ^®^, the main component of the lipid matrix of the NLC-DOX. The endothermic peak of Compritol 888 ATO ^®^ remains present in the DSC thermogram of NLC-DOX, providing evidence that the lipid matrix of NLC-DOX remains solid at room temperature. Nevertheless, this endothermic peak is broader and has a lower melting temperature (63.85 °C) when compared to the pure Compritol 888 ATO ^®^ (69.79 °C). The broader peak and reduction of melting temperature of the solid lipid matrix is evidence of the interactions of formulations components and liquid lipids, resulting in a less ordered lattice arrangement of solid matrix [79,80]. The reduced melting point observed for the solid lipid matrix might also be due to the nanometric size of NLC-DOX, resulting in a high surface area [81,82]. The FTIR results corroborate the data obtained in the DSC analyzes. The NLC-DOX spectrum showed bands characteristic of Compritol 888 ATO ^®^ and some bands of DOX, but to a lesser extent. There are small differences in the absorption regions, suggesting a greater affinity between DOX and lipid matrix.

An important observation is that the developed NLC-DOX presented a controlled and sustained release of DOX during the 72 h of the study. While the free DOX released approximately 80% of DOX in the first 4 h, at the same period of time, the NLC-DOX released less than 20%. The in vitro release kinetics evaluation of NLC-DOX followed the zero-order kinetic model (R^2^ = 0.9862). A zero-order kinetic model was also described for other authors who used NLCs to load different drug models [83,84]. However, none of them described the development of an NLC-DOX with a controlled and sustained release obeying a zero-order model. The zero-order model describes a constant release of payloads, which is ideal to achieve the desired pharmacological action with reduced side effects [64]. These controlled-release data also reflect the high encapsulation efficiency, drug loading and affinity observed in the characterization studies, suggesting that DOX is distributed throughout the solid lipid matrix of NLC. Then, the solid-lipid matrix of NLC serves as a physical barrier to the aqueous environment and the DOX it is not available to be immediately released in the receptor fluid, being released in a controlled way.

It is interesting to note that, although the encapsulation of DOX in NLC using the ion pair strategy can allow a controlled drug release at pH 7.4, it can be different in the tumor microenvironment. In other studies of our group different formulations of NLC-DOX, produced with different counter ions, showed that NLC-DOX is more efficient in reaching toxic levels of DOX in tumor cells, when compared to free DOX [33,56]. This occurs because, in the acid environment of the tumor area [85], there is an increase in the protonation of the COOH group of OA and a consequent decrease in the ion pair interaction, facilitating the release of DOX in the tumor microenvironment [71].

Considering that mucositis is one of the most serious and limiting complications of chemotherapy treatments, we aimed to evaluate if the encapsulation of DOX in an NLC matrix could provide a reduction of the mucositis process. In contrast to free DOX, NLC-DOX caused mild weight loss, one important sign of less severe mucositis [86], suggesting a more tolerable drug presentation. This can be associated with the different pattern of drug release observed for free DOX in comparison to the NLC-DOX. Since NLC-DOX provided controlled release of DOX, the impact of the drug toxicity possibly was less pronounced, allowing intestinal adjustment time.

Mucositis is characterized by villus atrophy, enterocyte damage and inflammatory cell infiltration in intestinal mucosa [6,87], which were observed in the free DOX treated group. The NLC-DOX group, on the other hand, showed preserved villus integrity and less inflammatory infiltrate compared to the free DOX group. The loss of the intestinal integrity induced by chemo and radiotherapy can increase intestinal permeability, allowing bacterial translocation and causing severe infection on the patients [88,89,90,91,92]. We observed that NLC-DOX treated animals showed preserved intestinal permeability. Importantly, tissue cohesion is driven by the tight junctions, proteins that promote strong cell–cell adhesion, which are important players in preserving intestinal epithelial monolayer integrity [93,94]. The NLC-DOX displayed increased expression of the two major tight junctions in the small intestine, Occludin and ZO1. We speculate that the controlled release of the drug allowed for better adjustment of the intestine to the DOX toxic mechanisms.

The expression of cytokines (inflammatory and homeostatic) associated with mucositis in the ileum of mice were also evaluated. We analyzed cytokines associated with the type 2 proinflammatory response (IL-4, IL-5, IL-13, IL-25, IL-33 and TSLP) and the cytokines involved in tissue regeneration and intestine homeostasis (IL-9 and Amphiregulin) [95,96]. Surprisingly, in the animals treated with the free DOX, an inhibition of expression of IL-4, IL-13, TSLP and IL-9 was observed in comparison to the saline group (*p* < 0.05). These results are probably associated with the intense degree of local tissue damage during the peak of tissue lesions [97]. In the animals treated with the NLC-DOX was observed the same pattern of cytokine expression in comparison to saline group (*p* > 0.5), except for IL-25 (*p* < 0.05), where a higher expression was detected. This result is in accordance with the morphology results. Since the treatment with NLC-DOX limited the mucositis process, the pattern of cytokines expression was maintained. Finally, although no difference was observed between free DOX group and NLC-DOX group for IL-9 (*p* > 0.5), a higher expression of Amphiregulin was observed for NLC-DOX group (*p* < 0.05), showing a higher ability to improve the tissue regeneration, even with a lower tissue damage. In the intestinal epithelium, cytokines such as IL-9 and Amphiregulin are involved with tissue regeneration [98,99,100,101]. Therefore, the encapsulation of DOX in NLC cannot only be able to improve the efficacy, as shown in different studies [27,67], but can also reduce the mucositis, which is an important adverse effect of DOX treatment.

## 5. Conclusions

The developed NLC-DOX showed spherical particles with size, PDI and Zeta potential adequate for parenteral administration, as well as high levels of DOX encapsulation and drug loading. The DSC and FTIR analyses showed a high interaction of DOX with the lipid matrix of NLC, which are in accordance with the high encapsulation and drug loading observed, resulting in a controlled drug release and, finally, preventing mucositis in the murine model. These results suggest that the developed NLC-DOX can enhance the safety of the treatment and, consequently, the quality of life of the patient.

## Figures and Tables

**Table 1:** Nucleotide sequence of the primers used.

Gene	Primer Forward	Primer Reverse
ZO-1	CCAGCTTATGAAAGGGTTGTTC	TCCTCTCTTGCCAACTTTTCTC
Occludin	ATGTCCGGCCGATGCTCTC	TTTGGCTGCTCTTGGGTCTGTAT
IL-33	ATTTCCCCGGCAAAGTTCAG	AACGGAGTCTCATGCAGTAGA
TSLP	ACGGATGGGGCTAACTTACAA	AGTCCTCGATTTGCTCGAACT
IL-4	GGTCTCAACCCCCAGCTAGT	GCCGATGATCTCTCTCAAGTGAT
IL-13	CCTGGCTCTTGCTTGCCTT	GGTCTTGTGTGATGTTGCTCA
IL-22	ATGAGTTTTTCCCTTATGGGGAC	GCTGGAAGTTGGACACCTCAA
IL-23	ATGCTGGATTGCAGAGCAGTA	ACGGGGCACATTATTTTTAGTCT
IL-5	CTCTGTTGACAAGCAATGAGACG	TCTTCAGTATGTCTAGCCCCTG
IL-25	ACAGGGACTTGAATCGGGTC	TGGTAAAGTGGGACGACGGAGTTG
GAPDH	AGGTCGGTGTGAACGGATTTG	TGTAGACCATGTAGTTGAGGTCA

## Data Availability

Data are contained within the article.

## References

[1] (2020). WHO Report on Cancer: Setting Priorities, Investing Wisely and Providing Care for All.

[2] Liu D., Liu Z., Wang L., Zhang C., Zhang N. (2011). Nanostructured lipid carriers as novel carrier for parenteral delivery of docetaxel. Colloids Surf. B Biointerfaces Sci..

[3] Fernandes E., Ferreira D., Peixoto A., Freitas R., Relvas-Santos M., Palmeira C., Martins G., Barros A., Santos L.L., Sarmento B. (2019). Glycoengineered nanoparticles enhance the delivery of 5-fluoroucil and paclitaxel to gastric cancer cells of high metastatic potential. Int. J. Pharm..

[4] Awasthi R., Roseblade A., Hansbro P.M., Rathbone M.J., Dua K., Bebawy M. (2018). Nanoparticles in Cancer Treatment: Opportunities and Obstacles. Curr. Drug Targets.

[5] Elting L.S., Cooksley C., Chambers M., Cantor S.B., Manzullo E., Rubenstein E.B. (2003). The burdens of cancer therapy. Clinical and economic outcomes of chemotherapy-induced mucositis. Cancer.

[6] Sonis S.T., Elting L.S., Keefe D., Peterson D.E., Schubert M., Hauer-Jensen M., Bekele B.N., Raber-Durlacher J., Donnelly J.P., Rubenstein E.B. (2004). Perspectives on cancer therapy-induced mucosal injury: Pathogenesis, measurement, epidemiology, and consequences for patients. Cancer.

[7] Pulito C., Cristaudo A., La Porta C., Zapperi S., Blandino G., Morrone A., Strano S. (2020). Oral mucositis: The hidden side of câncer therapy. J. Exp. Clin. Cancer Res..

[8] Lai H.C., Yeh Y.C., Ting C.T., Lee W.L., Lee H.W., Wang L.C., Wang K.Y., Lai H.C., Wu A., Liu T.J. (2010). Doxycycline suppresses doxorubicin-induced oxidative stress and cellular apoptosis in mouse hearts. Eur. J. Pharmacol..

[9] Ma P., Mumper R.J. (2013). Anthracycline Nano-Delivery Systems to Overcome Multiple Drug Resistance: A Comprehensive Review. Nano Today.

[10] Chaikomon K., Chattong S., Chaiya T., Tiwawech D., Sritana-Anant Y., Sereemaspun A., Manotham K. (2018). Doxorubicin-conjugated dexamethasone induced MCF-7 apoptosis without entering the nucleus and able to overcome MDR-1-induced resistance. Drug Des. Dev. Ther..

[11] Tacar O., Sriamornsak P., Dass C.R. (2013). Doxorubicin: An update on anticancer molecular action, toxicity and novel drug delivery systems. J. Pharm. Pharmacol..

[12] Goto S., Ihara Y., Urata Y., Izumi S., Abe K., Koji T., Kondo T. (2001). Doxorubicin-induced DNA intercalation and scavenging by nuclear glutathione S-transferase π. FASEB J..

[13] Bodley A., Liu L.F., Israel M., Seshadri R., Koseki Y., Giuliani F.C., Kirschenbaum S., Silber R., Potmesil M. (1989). DNA Topoisomerase II-mediated Interaction of Doxorubicin and Daunorubicin Congeners with DNA. Cancer Res..

[14] Asensio-López M.C., Soler F., Sánchez-Más J., Pascual-Figal D., Fernández-Belda F., Lax A. (2016). Early oxidative damage induced by doxorubicin: Source of production, protection by GKT137831 and effect on Ca2+ transporters in HL-1 cardiomyocytes. Arch. Biochem. Biophys..

[15] McCullough R.W. (2017). US oncology-wide incidence, duration, costs and deaths from chemoradiation mucositis and antimucositis therapy benefits. Future Oncol..

[16] Lipshultz S.E., Cochran T.R., Franco V.I., Miller T.L. (2013). Treatment-related cardiotoxicity in survivors of childhood cancer. Nat. Rev. Clin. Oncol..

[17] Shi Y., Moon M., Dawood S., McManus B., Liu P.P. (2011). Mechanisms and management of doxorubicin cardiotoxicity. Herz.

[18] Raber-Durlacher J.E., Weijl N.I., Abu Saris M., de Koning B., Zwinderman A.H., Osanto S. (2000). Oral mucositis in patients treated with chemotherapy for solid tumors: A retrospective analysis of 150 cases. Support Care Cancer.

[19] Kaczmarek A., Brinkman B.M., Heyndrickx L., Vandenabeele P., Krysko D.V. (2012). Severity of doxorubicin-induced small intestinal mucositis is regulated by the TLR-2 and TLR-9 pathways. J. Pathol..

[20] Elad S., Cheng K.K.F., Lalla R.V., Yarom N., Hong C., Logan R.M., Bowen J., Gibson R., Saunders D.P., Zadik Y. (2020). MASCC/ISOO clinical practice guidelines for the management of mucositis secondary to cancer therapy. Cancer.

[21] Kucharczyk K., Florczak A., Deptuch T., Penderecka K., Jastrzebska K., Mackiewicz A., Dams-Kozlowska H. (2020). Drug affinity and targeted delivery: Double functionalization of silk spheres for controlled doxorubicin delivery into Her2-positive cancer cells. J. Nanobiotechnol..

[22] Li J., Li X., Liu P. (2020). Doxorubicin-doxorubicin conjugate prodrug as drug self-delivery system for intracellular pH-riggered slow release. Colloids Surf. B Biointerfaces.

[23] Oladipo A.O., Nkambule T.T.I., Mamba B.B., Msagati T.A.M. (2020). The stimuli-responsive properties of doxorubicin adsorbed onto bimetallic Au@Pd nanodendrites and its potential application as drug delivery platform. Mater. Sci. Eng. C.

[24] Wang C., Qi P., Lu Y., Liu L., Zhang Y., Sheng Q., Wang T., Zhang M., Wang R., Song S. (2020). Bicomponent polymeric micelles for pH-controlled delivery of doxorubicin. Drug Deliv..

[25] Sedlacek O., Driessche A.V., Uvyn A., Geest B.G.D., Hoogenboom R. (2020). Poly(2-methyl-2-oxazoline) conjugates with doxorubicin: From synthesis of high drug loading water-soluble constructs to in vitro anti-cancer properties. J. Control. Release.

[26] Fraix A., Conte C., Gazzano E., Riganti C., Quaglia F., Sortino S. (2020). Overcoming Doxorubicin Resistance with Lipid-Polymer Hybrid Nanoparticles Photoreleasing Nitric Oxide. Mol. Pharm..

[27] Mussi S.V., Sawant R., Perche F., Oliveira M.C., Azevedo R.B., Ferreira L.A.M., Torchilin V.P. (2014). Novel Nanostructured Lipid Carrier Co-Loaded with Doxorubicin and Docosahexaenoic Acid Demonstrates Enhanced in Vitro Activity and Overcomes Drug Resistance in MCF-7/Adr Cells. Pharm. Res..

[28] Zhao S., Minh L.V., Li N., Garamus V.M., Handge U.A., Liu J., Zou A. (2016). Doxorubicin hydrochloride-oleic acid conjugate loaded nanostructured lipid carriers for tumor specific drug release. Colloids Surf. B Biointerfaces.

[29] Popilski H., Feinshtein V., Kleiman S., Mattarei A., Garofalo M., Salmaso S., Stepensky D. (2021). Doxorubicin liposomes cell penetration enhancement and its potential drawbacks for the tumor targeting efficiency. Int. J. Pharm..

[30] Makwana V., Karanjia J., Haselhorst T., Anoopkumar-Dukie S., Rudrawar S. (2021). Liposomal doxorubicin as targeted delivery platform: Current trends in surface functionalization. Int. J. Pharm..

[31] Perez A.T., Domenech G.H., Frankel C., Vogel C.L. (2002). Pegylated liposomal doxorubicin (Doxil) for metastatic breast cancer: The Cancer Research Network, Inc., experience. Cancer Investig..

[32] Leonard R.C.F., Williams S., Tulpule A., Levine A.M., Oliveros S. (2009). Improving the therapeutic index of anthracycline chemotherapy: Focus on liposomal doxorubicin (Myocet™). Breast.

[33] Lages E.B., Fernandes R.S., Silva J.O., de Souza Â.M., Cassali G.D., de Barros A.L.B., Ferreira L.A.M. (2020). Co-delivery of doxorubicin, docosahexaenoic acid, and α-tocopherol succinate by nanostructured lipid carriers has a synergistic effect to enhance antitumor activity and reduce toxicity. Biomed. Pharmacother..

[34] Dingler A., Gohla S. (2002). Production of solid lipid nanoparticles (SLN): Scaling up feasibilities. J. Microencapsul..

[35] Mu H., Holm R. (2018). Solid lipid nanocarriers in drug delivery: Characterization and design. Expert Opin Drug Deliv..

[36] Haider M., Abdin S.M., Kamal L., Orive G. (2020). Nanostructured Lipid Carriers for Delivery of Chemotherapeutics: A Review. Pharmaceutics.

[37] Shidhaye S.S., Vaidya R., Sutar S., Patwardhan A., Kadam V.J. (2008). Solid lipid nanoparticles and nanostructured lipid carriers--innovative generations of solid lipid carriers. Curr. Drug Deliv..

[38] Müller R.H., Radtke M., Wissing S.A. (2002). Solid lipid nanoparticles (SLN) and nanostructured lipid carriers (NLC) in cosmetic and dermatological preparations. Adv. Drug Deliv. Rev..

[39] Wu M., Fan Y., Lv S., Xiao B., Ye M., Zhu X. (2016). Vincristine and temozolomide combined chemotherapy for the treatment of glioma: A comparison of solid lipid nanoparticles and nanostructured lipid carriers for dual drugs delivery. Drug Deliv..

[40] Zhang J., Xiao X., Zhu J., Gao Z. (2018). Lactoferrin- and RGD-comodified, temozolomide and vincristine-coloaded nanostructured lipid carriers for gliomatosis cerebri combination therapy. Int. J. Nanomed..

[41] Mussi S.V., Silva R.C., Oliveira M.C., Lucci C.M., Azevedo R.B., Ferreira L.A. (2013). New approach to improve encapsulation and antitumor activity of doxorubicin loaded in solid lipid nanoparticles. Eur. J. Pharm. Sci..

[42] Fulop Z., Gref R., Loftsson T. (2013). A permeation method for detection of self-aggregation of doxorubicin in aqueous environment. Int. J. Pharm..

[43] Battaglia L., Gallarate M., Peira E., Chirio D., Muntoni E., Biasibetti E., Capucchio M.T., Valazza A., Panciani P.P., Lanotte M. (2014). Solid lipid nanoparticles for potential doxorubicin delivery in glioblastoma treatment: Preliminary in vitro studies. J. Pharm. Sci..

[44] Ma P., Dong X., Swadley C.L., Gupte A., Leggas M., Ledebur H.C., Mumper R.J. (2009). Development of idarubicin and doxorubicin solid lipid nanoparticles to overcome Pgp-mediated multiple drug resistance in leukemia. J. Biomed. Nanotechnol..

[45] Oliveira M.S., Mussi S.V., Gomes D.A., Yoshida M.I., Frezard F., Carregal V.M., Ferreira L.A.M. (2016). α-Tocopherol Succinate Improves Encapsulation and Anticancer Activity of Doxorubicin Loaded in Solid Lipid Nanoparticles. Colloids Surf. B Biointerfaces Sci..

[46] Subedi R.K., Kang K.W., Choi H.K. (2009). Preparation and characterization of solid lipid nanoparticles loaded with doxorubicin. Eur. J. Pharm. Sci..

[47] Miglietta A., Cavalli R., Bocca C., Ludovica G., Gasco M.R. (2000). Cellular uptake and cytotoxicity of solid lipid nanospheres (SLN) incorporating doxorubicin or paclitaxel. Int. J. Pharm..

[48] Zara G.P., Cavalli R., Bargoni A., Fundarò A., Vightto D., Gasco M.R. (2002). Intravenous administration to rabbits of non-stealth and stealth doxorubicin-loaded solid lipid nanoparticles at increasing concentrations of stealth agent: Pharmacokinetics and distribution of doxorubicin in brain and other tissues. J. Drug Target.

[49] Steiniger S.C., Kreuter J., Khalansky A.S., Sckidan I.N., Bobruskin A.I., Smirnova Z.S., Severin S.E., UHL R., Kock M., Geiger K.D. (2004). Chemotherapy of glioblastoma in rats using doxorubicin-loaded nanoparticles. Int. J. Cancer.

[50] Wong H.L., Rauth A.M., Bendayan R., Manias J.L., Ramaswamy M., Liu Z., Erhan S.Z., Wu X.Y. (2006). A new polymer-lipid hybrid nanoparticle system increases cytotoxicity of doxorubicin against multidrug-resistant human breast cancer cells. Pharm. Res..

[51] Zhang X., Sun X., Li J., Zhang X., Gong T., Zhang Z. (2011). Lipid nanoemulsions loaded with doxorubicin-oleic acid ionic complex: Characterization, in vitro and in vivo studies. Pharmazie.

[52] Siddiqui A., Gupta V., Liu Y.Y., Nazzal S. (2012). Doxorubicin and MBO-asGCS oligonucleotide loaded lipid nanoparticles overcome multidrug resistance in adriamycin resistant ovarian cancer cells (NCI/ADR-RES). Int. J. Pharm..

[53] Chen H.H., Huang W.C., Chiang W.H., Liu T.I., Shen M.Y., Hsu Y.H., Lin S.C., Chiu H.C. (2015). pH-Responsive therapeutic solid lipid nanoparticles for reducing P-glycoprotein-mediated drug efflux of multidrug resistant cancer cells. Int. J. Nanomed..

[54] Colas S., Maheo K., Denis F., Goupille C., Hoinard C., Champeroux P., Tranquart F., Bougnoux P. (2006). Sensitization by dietary docosahexaenoic acid of rat mammary carcinoma to anthracycline: A role for tumor vascularization. Clin. Cancer Res..

[55] Fundaro A., Cavalli R., Bargoni A., Vighetto D., Zara G.P., Gasco M.R. (2000). Non-stealth and stealth solid lipid nanoparticles (SLN) carrying doxorubicin: Pharmacokinetics and tissue distribution after i.v. administration to rats. Pharmacol. Res..

[56] Borges G.S.M., Silva J.O., Fernandes R.S., de Souza Â.M., Cassali G.D., Yoshida M.I., Leite E.A., de Barros A.L.B., Ferreira L.A.M. (2019). Sclareol is a potent enhancer of doxorubicin: Evaluation of the free combination and co-loaded nanostructured lipid carriers against breast cancer. Life Sci..

[57] Dash S., Murthy P.N., Nath L., Chowdhury P. (2010). Kinetic modeling on drug release from controlled drug delivery systems. Acta Pol. Pharm..

[58] Lu T., Ten Hagen T.L.M. (2017). Inhomogeneous crystal grain formation in DPPC-DSPC based thermosensitive liposomes determines content release kinetics. J. Control. Release.

[59] Santos R.G.C., Viana M.L., Generoso S.V., Arantes R.E., Correia M.I.T.D., Cardoso V.N. (2010). Glutamine supplementation decreases intestinal permeability and preserves gut mucosa integrity in an experimental mouse model. J. Parenter. Enter. Nutr..

[60] Rudra A., Deepa R.M., Ghosh M.K., Ghosh S., Mukherjee B. (2010). Doxorubicin-loaded phosphatidylethanolamine-conjugated nanoliposomes: In Vitro characterization and their accumulation in liver, kidneys, and lungs in rats. Int. J. Nanomed..

[61] Li S., Ma Y., Yue X., Cao Z., Dai Z. (2009). One-pot construction of doxorubicin conjugated magnetic silica nanoparticles. New J. Chem..

[62] Shah B.M., Khunt D., Bhatt H., Misra M., Padh H. (2016). Intranasal delivery of venlafaxine loaded nanostructured lipid carrier: Risk assessment and QbD based optimization. J. Drug Deliv. Sci. Technol..

[63] Kallakunta V.R., Tiwari R., Sarabu S., Bandari S., Repka M.A. (2018). Effect of formulation and process variables on lipid based sustained release tablets via continuous twin screw granulation: A comparative study. Eur. J. Pharm. Sci. Off. J. Eur. Fed. Pharm. Sci..

[64] Costa P., Sousa Lobo J.M. (2001). Modeling and comparison of dissolution profiles. Eur. J. Pharm. Sci..

[65] Von Moltke J., Ji M., Liang H.E., Locksley R.M. (2016). Tuft-cell-derived IL-25 regulates an intestinal ILC2-epithelial response circuit. Nature.

[66] Pallerla S., Gauthier T., Sable R., Jois S.D. (2017). Design of a doxorubicin-peptidomimetic conjugate that targets HER2-positive cancer cells. Eur. J. Med. Chem..

[67] Fernandes R.S., Silva J.O., Mussi S.V., Lopes S.C.A., Leite E.A., Cassali G.D., Cardoso V.N., Townsend D.M., Colletti P.M., Ferreira L.A.M. (2018). Nanostructured Lipid Carrier Co-loaded with Doxorubicin and Docosahexaenoic Acid as a Theranostic Agent: Evaluation of Biodistribution and Antitumor Activity in Experimental Model. Mol. Imaging Biol..

[68] Ni S., Qiu L., Zhang G., Zhou H., Han Y. (2017). Lymph cancer chemotherapy: Delivery of doxorubicin-gemcitabine prodrug and vincristine by nanostructured lipid carriers. Int. J. Nanomed..

[69] Tripathi C.B., Parashar P., Arya M. (2020). Biotin anchored nanostructured lipid carriers for targeted delivery of doxorubicin in management of mammary gland carcinoma through regulation of apoptotic modulator. J. Liposome Res..

[70] Wang Y., Zhang H., Hao J., Li B., Li M., Xiuwen W. (2016). Lung cancer combination therapy: Co-delivery of paclitaxel and doxorubicin by nanostructured lipid carriers for synergistic effect. Drug Deliv..

[71] Oliveira M.S., Lima B.H.S., Goulart G.A.C., Mussi S.V., Borges G.S.M., Oréfice R.L., Ferreira L.A.M. (2018). Improved Cytotoxic Effect of Doxorubicin by Its Combination with Sclareol in Solid Lipid Nanoparticle Suspension. J. Nanosci. Nanotechnol..

[72] Singh S., Lohani A., Mishra A.K., Verma A. (2018). Formulation and evaluation of carrot seed oil-based cosmetic emulsions. J. Cosmet. Laser Ther..

[73] Guilherme V.A., Ribeiro L.N.M., Alcântara A.C.S., Castro S.R., da Silva G.H.R., da Silva C.G., Breitkreitz M.C., Clemente-Napimoga J., Macedo C.G., Abdalla H.B. (2019). Improved efficacy of naproxen-loaded NLC for temporomandibular joint administration. Sci. Rep..

[74] Filipe V., Hawe A., Jiskoot W. (2010). Critical evaluation of Nanoparticle Tracking Analysis (NTA) by NanoSight for the measurement of nanoparticles and protein aggregates. Pharm. Res..

[75] Danaei M., Dehghankhold M., Ataei S., Hasanzadeh Davarani F., Javanmard R., Dokhani A., Khorasani S., Mozafar M.R. (2018). Impact of Particle Size and Polydispersity Index on the Clinical Applications of Lipidic Nanocarrier Systems. Pharmaceutics.

[76] Gao L., Li Q., Zhang J., Huang Y., Deng L., Li C., Tai G., Ruan B. (2019). Local penetration of doxorubicin via intrahepatic implantation of PLGA based doxorubicin-loaded implants. Drug Deliv..

[77] Tahir N., Madni A., Correia A., Rehman M., Balasubramanian V., Khan M.M., Santos H.A. (2019). Lipid-polymer hybrid nanoparticles for controlled delivery of hydrophilic and lipophilic doxorubicin for breast cancer therapy. Int. J. Nanomed..

[78] Li X., Jia X., Niu H. (2018). Nanostructured lipid carriers co-delivering lapachone and doxorubicin for overcoming multidrug resistance in breast cancer therapy. Int. J. Nanomed..

[79] Jenning V., Mäder K., Gohla S.H. (2000). Solid lipid nanoparticles (SLN) based on binary mixtures of liquid and solid lipids: A (1)H-NMR study. Int. J. Pharm..

[80] Castelli F., Puglia C., Sarpietro M.G., Rizza L., Bonina F. (2005). Characterization of indomethacin-loaded lipid nanoparticles by differential scanning calorimetry. Int. J. Pharm..

[81] Sakai H. (1996). Surface-induced melting of small particles. Surf. Sci..

[82] Antoniammal P., Arivuoli D. (2012). Size and Shape Dependence on Melting Temperature of Gallium Nitride Nanoparticles. J. Nanomater..

[83] Rahman H.S., Rasedee A., How C.W., Abdul A.B., Zeenathul N.A., Hemn H.O., Saeed M.I., Yeap S.K. (2013). Zerumbone-loaded nanostructured lipid carriers: Preparation, characterization, and antileukemic effect. Int. J. Nanomed..

[84] Shah N.V., Seth A.K., Balaraman R., Aundhia C.J., Maheshwari R.A., Parmar G.R. (2016). Nanostructured lipid carriers for oral bioavailability enhancement of raloxifene: Design and in vivo study. J. Adv. Res..

[85] Trédan O., Galmarini C.M., Patel K., Tannock I.F. (2007). Drug resistance and the solid tumor microenvironment. J. Natl. Cancer Inst..

[86] Meirovitz A., Kuten M., Billan S., Abdah-Bortnyak R., Sharon A., Peretz T., Sela M., Schaffer M., Barak V. (2010). Cytokines levels, Severity of acute mucositis and the need of PEG tube installation during chemo-radiation for head and neck cancer*—*A prospective pilot study. Radiat. Oncol..

[87] Lee C.S., Ryan E.J., Doherty G.A. (2014). Gastro-intestinal toxicity of chemotherapeutics in colorectal cancer: The role of inflammation. World J. Gastroenterol..

[88] Thomsen M., Vitetta L. (2018). Adjunctive Treatments for the Prevention of Chemotherapy- and Radiotherapy-Induced Mucositis. Integrative Cancer Therapies.

[89] Segers C., Mysara M., Claesen J., Baatout S., Leys N., Lebeer S., Verslegers M., Mastroleo F. (2021). Intestinal mucositis precedes dysbiosis in a mouse model for pelvic irradiation. ISME Commun..

[90] de Barros P.A.V., Andrade M.E.R., Generoso S.d.V., Miranda S.E.M., dos Reis D.C., Leocádio P.C.L., de Sales E Souza E.L., Martins F.d.S., da Gama M.A.S., Cassali G.D. (2018). Conjugated linoleic acid prevents damage caused by intestinal mucositis induced by 5-fluorouracil in an experimental model. Biomed. Pharmacother..

[91] van Vliet M.J., Harmsen H.J.M., de Bont E.S.J.M., Tissing W.J.E. (2010). The Role of Intestinal Microbiota in the Development and Severity of Chemotherapy-Induced Mucositis. PLoS Pathog..

[92] Tulkens J., Vergauwen G., Van Deun J., Geeurickx E., Dhondt B., Lippens L., Scheerder M.A., Miinalainen I., Rappu P., Geest B.G. (2018). Increased levels of systemic LPS-positive bacterial extracellular vesicles in patients with intestinal barrier dysfunction. Gut.

[93] Groschwitz K.R., Hogan S.P. (2009). Intestinal barrier function: Molecular regulation and disease pathogenesis, *J*. Allergy Clin. Immunol..

[94] Wu T.K., Lim P.S., Jin J.S., Wu M.Y., Chen C.H. (2018). Impaired Gut Epithelial Tight Junction Expression in Hemodialysis Patients Complicated with Intradialytic Hypotension. BioMed Res. Int..

[95] Licona-Limón P., Henao-Mejia J., Temann A.U., Gagliani N., Licona-Limón I., Ishigame H., Hao L., Herbert D.B.R., Flavell R.A. (2013). Th9 Cells Drive Host Immunity against Gastrointestinal Worm Infection. Immunity.

[96] Wynn T. (2015). Type 2 cytokines: Mechanisms and therapeutic strategies. Nat. Rev. Immunol..

[97] Sonis S.T. (2004). Pathobiology of mucositis. Semin. Oncol. Nurs..

[98] Zaiss D.M.W., Gause W.C., Osborne L.C., Artis D. (2015). Emerging Functions of Amphiregulin in Orchestrating Immunity, Inflammation, and Tissue Repair. Immunity.

[99] Turner J.E., Morrison P.J., Wilhelm C., Wilson M., Ahlfors H., Renauld J.C., Panzer U., Helmby H., Stockinger B. (2013). IL-9-mediated survival of type 2 innate lymphoid cells promotes damage control in helminth-induced lung inflammation. J. Exp. Med..

[100] Chen F., Liu Z., Wu W., Rozo C., Bowdridge S., Millman A., Rooijen N.V., Urban J.F., Wynn T.A., Gause W.C. (2012). An essential role for TH2-type responses in limiting acute tissue damage during experimental helminth infection. Nat. Med..

[101] Monticelli L.A., Osborne L.C., Noti M., Tran S.V., Zaiss D.M.W., Artis D. (2015). IL-33 promotes an innate immune pathway of intestinal tissue protection dependent on amphiregulin–EGFR interactions. Proc. Natl. Acad. Sci. USA.

